# A cultural and political difference: comparing the racial and social framing of population crack cocaine use between the United States and France

**DOI:** 10.1186/s12954-022-00625-5

**Published:** 2022-05-12

**Authors:** Andrew Goulian, Marie Jauffret-Roustide, Sayon Dambélé, Rajvir Singh, Robert E. Fullilove

**Affiliations:** 1 Columbia University, Mailman School of Public Health, New York, USA; 2 CEMS, Inserm U1276, CNRS UMR 8044, EHESS, Paris, France; 3 British Columbia Centre on Substance Use (BCCSU), Vancouver, Canada; 4 Baldy Center for Law and Social Policy, Buffalo University of Social Sciences, New York, USA; 5 Institut Convergences Migrations, Campus Condorcet, Aubervilliers, France; 6 Agence Nationale de L’Inclusion Économique et Sociale, Conakry, Guinea; 7 Rural Workforce Agency Victoria, Melbourne, Australia

**Keywords:** Crack cocaine, Harm reduction, Racial issues, Structural vulnerability, Drug policy, Population health

## Introduction

Crack cocaine refers to a derivative of powder cocaine. Nonetheless, the usage of the term “crack cocaine” is loaded with unfortunate connotations of stigma and discrimination. When comparing the framing of crack cocaine use between the United States (U.S.) and France, the respective cultural and sociopolitical settings need to be accounted for.

In the U.S., crack cocaine refers to a smokable variety of cocaine which is considered to be more affordable and accessible than powder cocaine [1]. In the 1980s and 90 s, the U.S. response to crack cocaine was driven by media depictions of an urban, public health crisis primarily affecting black communities in American cities. This media depiction drove U.S. drug policy and shaped both political debate and public attitude towards crack cocaine [1, 2]. The subsequent influx of drug education messages, public service announcements, and curriculums that were created in response to crack cocaine were pervaded by the public and political fear that crack cocaine was destroying a generation of young Americans [3]. The U.S. government response at this time focused on managing the perceived crack cocaine epidemic by criminalizing rather than providing treatment facilities or healthcare services for people who use crack cocaine. In fact, the 1988 National Household Survey on Drug Abuse showed that the sharpest rise in cocaine and crack cocaine use since the inception of the survey in the early 1970s, rapidly outpaced the availability of treatment programs and efforts to expand treatment facilities in metropolitan areas such as New York City [4].

The French approach to crack cocaine shares commonalities and differences with the U.S. In France, crack cocaine is mostly smoked but more recently is also being injected intravenously and the spike of increased crack cocaine use in France did not occur until the 2000s [5, 6]. French political and media attention towards crack cocaine has also recently increased due to the presence of visible, open drug use in Paris framing the topic as a public order, health, and social problem. This attention stems from crack cocaine in France having increasing associations with socially and economically vulnerable populations when compared to powder cocaine use patterns [6]. Unlike the U.S., the aggregation of racial data is prohibited in France. And as a result, the French understanding of the current rise in crack cocaine use does not clearly analyze the issue along racial lines but rather considers low socioeconomic populations to be drivers of the recent increase in crack cocaine use [5]. Since the late 1970s, in addition to a repressive drug policy approach, France has implemented a strong, publicly funded drug treatment system with harm reduction services to better address the health needs of people who use drugs [7]. Accordingly, France is systematically more equipped to serve the healthcare needs of people who problematically use crack cocaine compared to the U.S. [5, 6].

## Framing of Crack Cocaine in the U.S.

In the U.S., crack cocaine is derived from powdered cocaine by combining it with water and another substance, usually bicarbonate soda. This substance is then heated and the newly formed solids broken into smaller pieces that are subsequently vaporized and inhaled [8]. The chemical alteration allows the end-user to purchase smaller amounts of crack cocaine at a lower cost, thereby increasing general accessibility.

Since the introduction of crack cocaine in American cities as a more accessible form of powder cocaine circa 1984, the U.S. response to the substance has been framed as an urbanized, racial issue that predominantly involves members of the Black community [1]. In the 1980s, the intersection of people who use crack cocaine and the HIV/AIDS epidemic propelled in the American psyche the idea that people who use crack cocaine in the U.S. is primarily associated with black communities in major metropolitan areas. During that time, as the prevalence of HIV was increasing in American cities, positive correlations were found between sexual risk-taking behaviors and crack cocaine use in black adolescent populations [9]. Other reports demonstrated how the popularity of crack cocaine significantly increased urban crime rates and boosted inner-city decay in major metropolitan areas of the U.S. in the late 1980s and early 1990s [10]. Meanwhile, mass media pedaled to the American public a fear-mongering, racist narrative of predominantly black “crack baby mothers” who risked burdening the U.S. healthcare system with an epidemic of “crack babies” [11].

The political framing of crack cocaine was exacerbated in 1986 when the death of young, Black basketball star Len Bias, who was widely presumed to be caused by overdose, received significant mass media coverage. As a result, Bias unwittingly became a public symbol of the dangers of crack cocaine. Bias’s death and surrounding media coverage were used as catalysts for significant drug policy changes, including the enactment of the U.S. Anti-Drug Abuse Act of 1986, which established minimum sentencing for possession of crack cocaine [12, 13]. And the resulting 100:1 sentencing disparity between crack and powder cocaine offenses was enforced despite the scientific community demonstrating that both powder and crack forms of cocaine produce identical and predictable physiological effects, negating any need for a difference in minimum sentencing [14].

The cultural framing about people who use crack cocaine have led to upholding American policies that continue to disproportionately target specific racial populations, particularly black communities. A 1993 JAMA publication confirmed that the prevalence of crack cocaine use did not depend on race-specific factors and showed that crack cocaine use did not differ significantly for African Americans or Hispanic Americans as compared to white Americans [15]. Additionally, Substance Abuse and Mental Health Administration reports data confirms there are no statistically significant differences in the rates of illicit drug use between racial and ethnic groups [16]. However, recent U.S. justice system data from the 2019 fiscal year shows an staggering 81.1% of smokable-cocaine trafficking offenders were black [17].

The perceived crack cocaine epidemic has currently subsided as a focal point in the American psyche, partly in reaction to significant neuroscientific data which refuted the false assumptions that one-time use of crack cocaine resulted in instant addiction issues [18]. Despite the media attention of the late 1980s and 90 s focusing on the negative effects of crack cocaine, this did not spur increased harm reduction efforts or drug treatment facilities. Data shows only roughly ten percent of the population who qualify for drug treatment ever receiving care for problematic drug use in the U.S. [16].

## Framing of Crack Cocaine in France

Unlike the U.S. where crack cocaine is predominantly with bicarbonate soda, in France, crack cocaine is also derived from cocaine powder cooked with ammonia that take the form of “rocks” ready to use by smoking or injecting routes, named “crack”. Crack cocaine is sometimes further prepared with bicarbonate soda forming a freebase crack cocaine variety. The minor differences in preparing and labeling terminology of different subtypes of crack cocaine are considered to be social distinguishers  [19].

While the presence of crack cocaine was first documented in France in 1986 by arrest record data from the Office Central du Trafic et de Répression des Stupéfiants and it wasn’t until the mid-1990s that ethnographic data first described crack cocaine use in public spaces in Paris [20, 21]. The general framing of crack cocaine in France is characterized by both an intersection of open drug use in public spaces with crack cocaine’s associations with low socioeconomic communities. In France, illicit drug use was criminalized by the Law of December 31st, 1970, which is also included in the Public Health Law Code [7]. France can be considered a paradox because the country has a repressive approach to drug policy, quite like the U.S., but at the same time has implemented a strong, publicly funded drug treatment system in the 1970–1980s followed by harm reduction services to better address the health needs of people who use drugs since the 1990s [7]. Since the onset of the HIV/AIDS epidemic, France has largely focused efforts on accessibility to harm reduction by focusing on drug treatment for opioid use without a strong effort to support people experiencing problematic crack cocaine use even if there are intersections between stimulants use and opioid substitutive treatments use [22].

Notably, in France, crack cocaine has not been framed as a public health epidemic, even with the trend of crack cocaine use and the rise of open drug scenes in Paris and its suburbs [6]. The origins of the French response to people who use crack cocaine can be traced to a spike of increased crack cocaine use in France in the early the 2000s with data showing foci in Paris, North-East suburbs, and overseas territories including French Guiana and Réunion [5]. More recent French public health surveys demonstrate crack cocaine is one of the most commonly used illicit substance, after cannabis, by people who engage with harm reduction services and drug treatment centers [23]. This substantial increase in the number of people who use crack cocaine led to the creation of harm reduction tools such as the dissemination of clean crack pipes. In 2019, in response to the increased visibility of open drug use in Paris and its suburbs in public spaces, the city of Paris, regional health agency, and Ile de France Prefecture implemented a “Crack Plan” to improve access to harm reduction services and social housing for people who use crack cocaine openly in Paris. The “Crack Plan” also proposed to strengthen the harm reduction services for people who use crack cocaine by increasing a public-funded healthcare model that is embedded in the French model of welfare state that strives to protect the most vulnerable populations [24]. Indeed, since 2020, access to housing for people who use crack cocaine living in precarious conditions has increased in Paris, thanks to the COVID-19 pandemic that paradoxically helped the harm reduction providers to transform this topic into a priority [25]. At the same time, a repressive policy towards crack cocaine open scenes and users who lived in these deprived areas is still present in France, especially in Paris and its suburbs where open drug scenes are visible. In September 2021, this repressive policy was symbolized by a wall that has been built to avoid people who use crack cocaine to move again to a residential area in the North-East of Paris [26].

In contrast with racial considerations in American policy, racial designations based on self-identification are not available in France.. Even though no statistical data on race is explicitly collected, the first ethnographic data about people who use crack cocaine in the early 1990s described them mainly as people from former French colonies in overseas territories, which mainly implies they are people of color [21]. To add, the racial crossroad is further reflected with the terminology of Parisian crack cocaine dealers referring to themselves as *modous* a word that stems from the Mouridi community from Senegal, as a product of past French colonization in West Africa [5]. Further qualitative ethnographic data demonstrates an intersection between people who use crack cocaine and migrants- another term that elicits proxy terminology and highlights racial and ethnic inequalities from postcolonial France [6, 27]. The French media depiction of crack cocaine often supposes a relationship between people who use crack cocaine and migrants, but it is interesting to note that migrants are often presented in media as victims of malevolent drug dealers [27, 28].

Importantly, the French framing of crack cocaine is not without a racial dimension as drug policy and the criminal justice system exhibit formidable ties. Discrimination and structural inequities regarding arrests and incarceration according to race have been documented by quantitative social science research [29]. Additionally, ethnographic studies have described an overrepresentation of people of color in French prisons coupled with a threefold increase in court appearances and higher arrest rates for foreign-born French nationals [30, 31]. The difficulties for highlighting explicitly racial inequities can be partially explained by the “ French Republic Universalism” prism that claims that all French people who belong to the same nation need to be considered as “equal citizens” with the “same rights” regardless of their origins. This framing brings about a color-blind political discourse that is unable to publicly recognize that discrimination against people of color according to the French colonialist ideology  [32].

## Commentary

The underlying American public attitude of crack cocaine as an urban, black problem has unfortunately contributed to widened racial health disparities. Rates of crack cocaine use have not been shown to be significantly higher between races, yet law enforcement efforts that target specific populations to prohibit the distribution of crack cocaine have resulted in the mass incarceration of black Americans [1]. Despite ethnographic data from France suggesting that racial and ethnic disparities exist, a direct comparison between U.S. and French drug policy and population crack cocaine use is not entirely applicable due to a lack of analogous data [21]. As a consequence of a color-blindness discourse, the French framing of people who use crack cocaine is primarily viewed through a social class lens.

In the U.S., despite successive waves of drug epidemics garnering significant media attention, drug treatment services are not readily available through publicly supported healthcare services for lower socioeconomic populations. Instead, American drug policy acts as a social determinant of health for minority populations who engage in crack cocaine use. In France, where racial justice is muted and conversations about ethnicity are entrenched in colonialist roots, a well-funded public healthcare system is available for people who use crack cocaine. In the French social context, people who use crack cocaine are considered as modern figures of precariousness in the social world of people who use drugs- comparable as previous “homeless heroin users” as described by Philippe Bourgois [33].

Both the U.S. and France have unique intersectionalities of culture, phenotype designations, language, and political boundaries all of which contribute to the framing of population crack cocaine use. A difference in social strategies in urban areas in both the U.S. and France where crack cocaine is distributed and consumed is reflected by the structural vulnerability of people who use crack cocaine use and the differences in political framings in both countries. In the U.S., the conversation about crack cocaine has focused on race, while the French discourse has been framed as a social and urban disparity problem [25, 26]. Nevertheless, policymakers in both nations have also stated concern about people who use crack cocaine in political terms favoring repressive responses that often leave out the possibility of social services and racial justice to address the underlying structural vulnerabilities [34].

## Data Availability

Not applicable.
